# The Chinese automobile industry’s research and development capability and innovative performance

**DOI:** 10.3389/fpsyg.2022.1093305

**Published:** 2023-01-05

**Authors:** Jili Liu, Guoxin Liu, Hainan Cui, Yuehua Chen

**Affiliations:** ^1^Business School, Hubei University, Wuhan, China; ^2^Hubei Center for Studies of Human Capital Development Strategy and Policy, Hubei University, Wuhan, China; ^3^School of Management, Wuhan University of Technology, Wuhan, China

**Keywords:** distributed innovation, innovation, research and development, automobile industry, fierce competition

## Abstract

The Report of China’s Automobile Industry Development 2016 points out that China’s automobile industry faces increasingly fierce competition from global peers, and domestic automobile manufacturers shall continuously improve independent innovation and industry innovative performance. Automobile manufacturers’ participation in distributed innovation and their research & development (R&D) capability play a key role in the growth of innovative performances of China’s automobile industry. With development of China’s automobile industry as the background, this study presents a theoretical framework of correlation of distributed innovation embedment, R&D capability and innovative performances of China’s automobile industry from the microscopic perspective of single industry. Several hypotheses were proposed and then verified by empirical method. Specifically, 117 effective questionnaires were collected through non-probability sampling and analyzed quantitatively, followed by hypothesis test. The results clarify the working principles of the three dimensions of distributed innovation embedment influencing innovative performances of China’s automobile industry and improvement of R&D capability of enterprises by distributed innovation. This study provides references to improvements of innovative performances of China’s automobile industry.

## 1. Introduction

Distributed innovation is a novel pattern of technological innovation that has been widely applied in various industries. Recently, distributed innovation embedment in enterprises have attracted great attention by scholars. The influences of distributed innovation on enterprises have been thoroughly investigated from the macroscopic perspective. It is believed that under the background of information explosion and intensified competition, distributed innovation embedment, which is characterized by cooperation, network, dynamics and sharing, can enhance innovation activities of enterprises and allow them to exploit comparative advantages, thus enhancing their performances. Yan (2016) claimed that distributed innovation can help multi-national corporations to adapt to local market by reducing the buffer period, promoting diffusion and sharing of knowledge and information, enhancing utilization efficiency of resources and lowering cost and risks of research & development (R&D). In this way, innovative performances and competitiveness of enterprises can be enhanced as Yan (2016). Nevertheless, distributed innovation still remains a topic with exciting research prospective. Appropriate distributed innovation embedment can bring considerable benefits to enterprises. As Baldwin (2012) highlighted that distributed innovation is not limited to the innovation from the manufacturer but also from other sources such as users and rivals. These multi driver of the innovation may give the competitive advantage and enhance the firm’s performance.

Gerybadze (2004) demonstrated that distributed innovation embedment was mostly applied in medicine, chemical industry, biotechnology and healthcare, communication equipment and services and information technology. Indeed, distributed innovation embedment has rarely been observed in automobile industry, which is a key part of national economy in developed countries. Indeed, the automobile industry is a direct indicator of national industrialization level. After the rapid growth over the past two decades, China’s automobile industry has attracted most top global players. However, China’s automobile industry has a big gap with top players in all aspects. This is reflected as old-fashioned innovative activities, low investment in R&D, few patents owned by enterprises, poor independent innovation and limited R&D capability. For this reason, the Twelfth and thirteenth Five-Year Plan of China emphasizes that the establishment of technological innovation systems with enterprises as the main player shall be accelerated, the reform of science and technology system shall be deepened, efficient allocation and integration of science and technology resources shall be promoted, enterprise innovations shall be motivated and corporations’ investment in R&D shall be encouraged. Therefore, distributed innovation embedment in strategy planning of China’s automobile manufacturers to enhance their core competitiveness and innovative performance are indeed imperative. The study is an effort to start the debate of distributed innovation in the Chinese automobile industry and empirically tests the role of distributed innovation on the financial performance of the firms. The study contributes in the existing literature in more than one way. First the study empirically tests the outcomes of distributed innovation in financial terms while existing literature discussed the it’s fit with existing innovation theories (Kelly, 2006), with logistics (Yuan, 2001; Zhao, 2012; Yan, 2013; Yang et al., 2016; Amling and Daugherty, 2020; Tang et al., 2022), with digital entrepreneurial opportunities (2022). Secondly the study enriches the existing literature by studying the fast-growing economy of China. Thirdly the study used the primary data rather than secondary data which may produce different results than secondary data. This study focuses on the correlation of R&D capability, distributed innovation embedment and innovative performances of China’s automobile industry. A correlation conceptual model of the three factors is established, relevant hypotheses are proposed, and empirical analysis and test are executed based on survey data of China’s automobile industry. It is demonstrated that application of knowledge collaboration, which is a dimension of distributed innovation embedment, in strategy planning tends to have positive effects on the R&D capability of China’s automobile industry and provide data support to R&D capability and innovative performance of China’s automobile industry during distributed innovation embedment.

The rest of the paper is organized as follow. The following section shed light on the theoretical background and hypothesis development. Section 3 discusses the research design, methodology and data. Section 4 presents the results and findings of the study. Section 5 concludes the study.

## 2. Theories and hypothesis

### 2.1. Innovative performance

The innovative performances of China’s automobile industry shall be evaluated by quantitative indexes of enterprises and qualitative indexes of managers. Hagedoorn and Cloodt (2003) proposed that innovative performances reflect all achievements by innovative activities and final values by strategy and R&D in a generalized and special way, respectively. In the same line knowledge-based view and absorptive capacity theory are utilized to judge the role of distributed innovation to the automobile industry. It is widely accepted that knowledge management from different sources on which distributed innovation dependent as different sources provide knowledge as users, suppliers resulted in innovation and competitive advantage as Flynn et al. (2003); Zack et al. (2009); Huang (2011); Lau and Bruton (2011); Jin et al. (2012); Kang et al. (2014); Iqbal et al. (2018); Qi and Chau (2018); Zand et al. (2018); Abubakar et al. (2019); Hammoud (2020). It is believed that this performance includes efficiency and performance of innovations. The innovative performances of China’s automobile industry can be evaluated in a subjective or objective way. The objective way emphasizes enterprise profit, including sales volume, return on investment and financial indexes, while the subjective way focuses on judgment on enterprise performance of the respondent, including evaluation of innovation capability and consumer satisfactory of new products. In either way, the conclusions are neither accurate nor convincing. Hence, some scholars believed that subjective and objective factors shall be combined to make the results convincing. We also believe that innovative performances of China’s automobile industry shall be evaluated based on both subjective and objective factors.

### 2.2. Correlation of distributed innovation embedment and innovative performance

Chesbrough et al. (2018) distribution innovation is the process of managing the flows of knowledge across the organizational boundaries using pecuniary and non-pecuniary mechanisms in line with the organizational business model. In the same line Bertello et al. (2022) stress the need of the distributed innovation for the firms to achieve the competitive advantage and survive in this competitive market. The study proposed that knowledge beyond the organizational boundaries influence the organizational performance. Cummings (2006) claimed that distributed innovations are achieved by execution of ideas, tasks or procedures by employees all over the world. Coombs and Metcalfe (2002) pointed out that distributed innovation refers to distributed realization of technologies and related capabilities required by innovation networks in different organizations and other knowledge production institutions. Owing to rapid globalization, researchers prefer to investigate distributed innovation from perspectives of era and resources. Dooley and O’Sullivan (2007) proposed that distributed innovation embedment lies within the entire supply chain of its organization and cooperative and project innovations induced by enterprise alliances have positive effects on enterprise performances such as innovations, thus enhancing its competitiveness in the global market. Yan et al. (2017) defined distributed innovation as collaborative innovation activities of enterprises during technological innovations. Specifically, enterprises achieve resource sharing and knowledge management by integrating internal and external resources in virtue of their advantages such as geographical dispersion. Liu et al. (2010) believed that distributed innovations are innovative activities by enterprises with cooperative relationship (e.g., upstream and downstream) based on resource sharing and shared network platform. Compared with centralized innovations, distributed innovations are characterized by resource sharing, regional distribution, simultaneity, coordination and cooperation, which enable excellent innovative performances of enterprises. Chen et al. (2017) divided distributed innovations into three dimensions: distributed structure, distributed cognition, distributed cooperation. These dimensions enable growth and profit of enterprises in various aspects. In other words, the positive effects of distributed innovation embedment on innovative performances of enterprises are strengthened.

Marlow et al. (2008) proposed the concept of knowledge collaboration and defined it as a process where multiple parties with knowledge resources participate in knowledge activities, with the aim of knowledge innovation. It is also proposed that knowledge collaboration is a dynamic process that can effectively enhance organizational competitiveness and innovative performance, thus achieving a collaborative effect for enterprises.

Previous studies of distributed innovation reveal that knowledge collaboration embedment has influences on innovative performances of enterprises. For instance, Hameed et al., (2018) examined the driver of the distributed innovation among the Malaysian small and medium enterprises and they concluded that R&D, external knowledge and internal innovation are the main drivers of the distributed innovation. Li et al. (2020) highlighted the effective use of organizational ambidexterity is significantly dependent upon the distributed innovation. They concluded that firms can only take the advantage of organizational ambidexterity is dependent how firms incorporate the knowledge outside the organizational boundaries. Oborn et al. (2019) highlighted the role of digital technologies in the development of distributed innovation and found that distributed innovation resulted in the better performance of the firms. With rapid development of knowledge economy, scholars divided distributed innovation into distributed structure, distributed cognition and knowledge collaboration. The distributed structure includes member distribution and project modularization within the distributed innovation network. The former refers to the geographical dispersion of members, while the latter enables close correlation of internal factors in the project with team task as the core part. The distributed cognition mainly refers to trust between members in the distributed network and similarity of distributed cognition. The knowledge collaboration consists of transfer and sharing of knowledge and innovation management, namely vertical and horizontal expansions of knowledge. Participation of China’s automobile enterprises in distributed innovation includes strengthening of geographic location and project modularization in distributed structure, strengthening of trust and cognitive inclination of members in distributed cognition, and strengthening of innovation management in knowledge collaboration, which is beneficial to their innovative performances. Despite its irreplaceable role in domestic economy, China’s automobile industry exhibits low overall competitiveness. Especially, China’s automobile manufacturers are significantly inferior to top players in the world in terms of innovative performances. Hence, significant improvements of innovative performances of China’s automobile industry are urgently needed. Distributed innovation embedment is beneficial to product innovation, management innovation and technological innovation in China’s automobile industry by reducing innovation cost and improving innovative performance.

In summary, the following hypotheses are proposed:

*Hypothesis H1*: Distributed innovation embedment has significant positive effects on innovative performances of China’s automobile industry.

*Hypothesis H1a*: Distributed structure embedment has significant positive effects on innovative performances of China’s automobile industry.

*Hypothesis H1b*: Distributed cognition embedment has significant positive effects on innovative performances of China’s automobile industry.

*Hypothesis H1c*: Knowledge collaborative embedment has significant positive effects on innovative performances of China’s automobile industry.

### 2.3. Correlation of distributed innovation embedment and R&D capability

Burgelman et al. (1996) claimed that technological innovation capability serves as the foundation of technological innovation strategy of enterprises. Leonard-Barton (2003) believed that technological innovation capability is a core competitiveness of enterprises. The innovation capability is essential for sustainable development of enterprises as it has positive effects on their innovative performances and competitiveness. Innovations are originated from R&D. Indeed, enhancement of enterprise innovation depends heavily on the improvement of its R&D capability. Hence, distributed innovation embedment in the R&D network plays a key role in enhancement of R&D capability.

Owing to limited resources and capabilities, enterprises have to rely on distributed innovation systems to enhance R&D capability. A distributed innovation system is both a collaborative process of different organizations within the distributed innovation network and an interactive process of mutual adaptation and interactions between these organizations and the external environment. The distributed structure of distributed innovation system delivers a R&D network with peers in the automobile industry or upstream/downstream enterprises by modularization of complex system and strategy alliance. In this way, the R&D capability of an enterprise can be strengthened. The distributed cognition of distributed innovation system emphasizes common interest-based distributed cognition and trust, as well as dependence of upstream and downstream enterprises, resulting in a large-scale economy of China’s automobile industry. The knowledge collaboration is indeed the core part of distributed innovation system. R&D is originated from collaboration of vertical processing and horizontal modularization of knowledge. Creativity, knowledge and technical guidance can be obtained from collaborations, resulting in an effective R&D system.

In summary, the following hypotheses are proposed:

*Hypothesis H2*: Distributed innovation embedment has significant positive effects on R&D capability of China’s automobile industry.

*Hypothesis H2a*: Distributed structure embedment has significant positive effects on R&D capability of China’s automobile industry.

*Hypothesis H1b*: Distributed cognition embedment has significant positive effects on R&D capability of China’s automobile industry.

*Hypothesis H1c*: Knowledge collaborative embedment has significant positive effects on R&D capability of China’s automobile industry.

### 2.4. Correlation of R&D capability performance

Drucker (2014) regarded enterprise innovation as a tool from the perspective of function. Specifically, innovation is regarded as a tool owned by enterprise and appropriate utilization of this tool inspires novel creativity from existing resources, thus achieving increased profits. R&D activities play a key role in enterprise competitiveness. However, Mr. Zhu Sen, the executive vice president of China Machinery Industry Federation, said that “most of the existing technologies in China’s automobile industry are imported, while independent R&D is still at an early stage.” Zhang et al. (2008) also insisted the dominant role of R&D capability in competitiveness of China’s automobile industry. Unfortunately, most investments by China’s automobile manufacturers have been used to expand the production capability instead of R&D, while foreign competitors have made considerable investments in R&D. In the market economy, enterprises are direct beneficiaries of R&D activities and R&D is supposed to attract great attention by enterprises. Enterprises’ investment in R&D serves as the objective foundation of ownership of core technologies by China’s automobile industry. Additionally, investment in R&D allows enterprises to surpass competitors in product innovation, technological innovation and management innovation, thus achieving high-end products and services.

In summary, the following hypothesis is proposed:

*Hypothesis H3*: R&D capability has significant positive effects on innovative performances of China’s automobile industry.

## 3. Research design and analysis

### 3.1. Scale design

**Figure 1:**
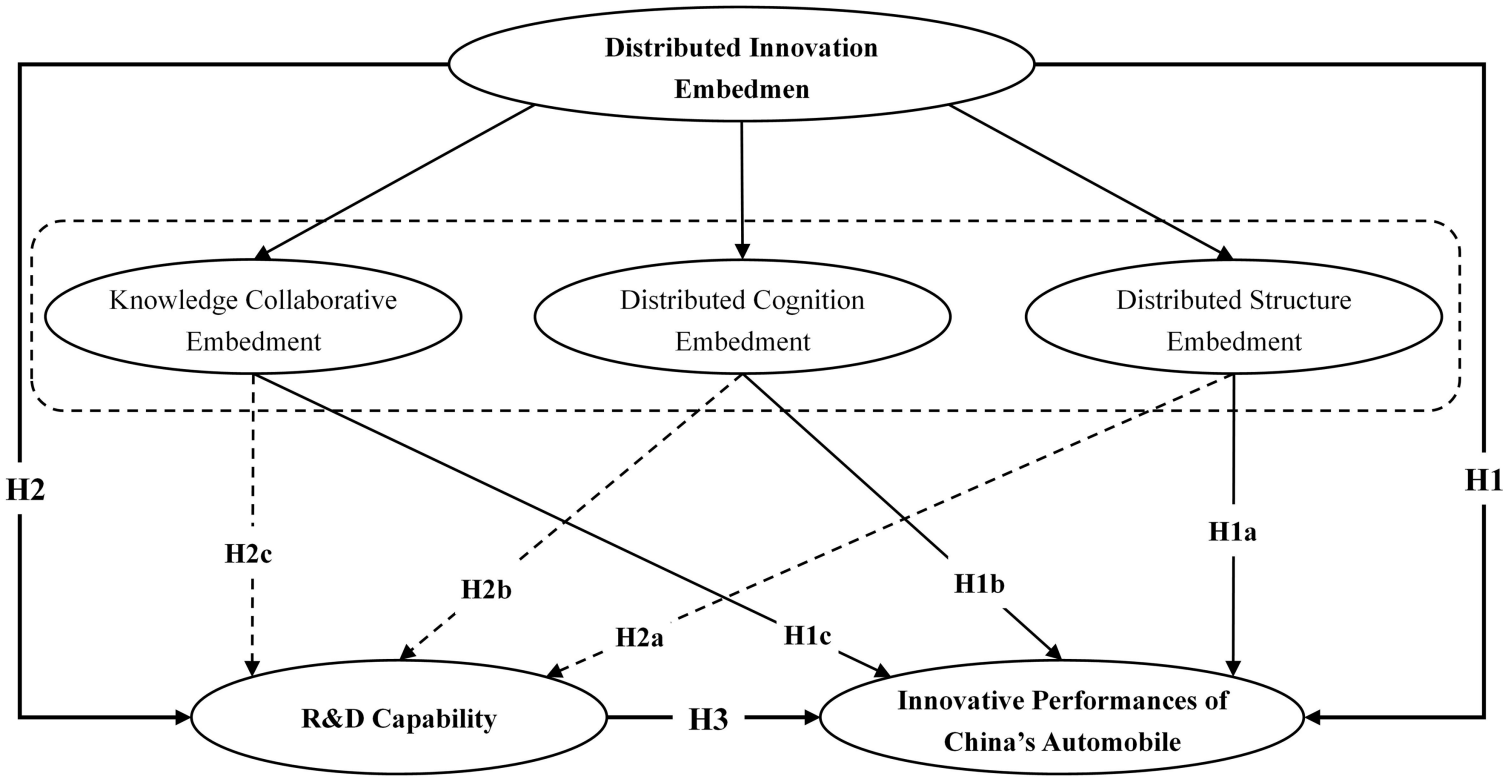
Proposed model.

The proposed model (see Figure 1) includes distributed innovation embedment (e.g., knowledge collaborative embedment, distributed cognition embedment, distributed structure embedment, R&D capability, innovative performances of China’s automobile industry). Questionnaires were designed and optimized according to professional suggestions. Each variable is reflected by several items and each item is evaluated by the Likert five-level scale (1 = significantly over-low, 5 = significantly over-high).

### 3.2. Variable measurement

Innovative performances of China’s automobile industry are affected by distributed innovation embedment, including knowledge collaborative. This study further explores the significance of distributed innovation (e.g., knowledge collaboration embedment, R&D capability) to enterprise development. Hence, innovative performances of China’s automobile industry, distributed innovation and R&D capability were measured. Innovative performances of China’s automobile industry have been investigated based on sales volume, market shares, financial indexes, evaluation of innovation capability by managers and consumer satisfactory on new products. Considering differences in strategy and management of enterprise innovation, we measured innovative performances of enterprises from perspectives of product innovation, product update, technological innovation and managers, which are denoted by IP1, IP2, IP3 and IP4, respectively. Six items were designed for this measurement as Tong (2011).

R&D capability was measured from perspectives of ratio of investment in R&D, patent quantities, R&D employees, which are denoted by RDA1, RDA2 and RDA3, respectively. Six items were designed for this measurement as Xie et al. (2013).

The variables of distributed innovation embedment were measured by methods reported elsewhere. Specifically, the variables are divided into categories of distributed structure embedment, distributed cognition embedment and knowledge collaborative embedment, which are denoted by DI1, DI2 and DI3, respectively. The distributed structure embedment focuses on geological distributions and project modularized allocation; the distributed cognition embedment focuses on trust, cognitive learning and cooperation among members; the knowledge collaborative embedment focuses on knowledge transfer, knowledge sharing and innovation management. Sixteen items were designed for this measurement as Li (2013).

### 3.3. Sample analysis

Questionnaires were designed according to the measurement variables and sent to representative automobile manufacturers in China, including FAW-Volkswagen, Dongfeng Motor Corporation, Dongfeng Peugeot Citroen Automobile Company Ltd. (DPCA), Renault S.A., Volvo and Dongfeng Yueda Kia (DYK). One hundred eighty-seven questionnaires were allocated *via* e-mail and 117 of them completed by respondents from automobile manufacturers participated in distributed innovation were regarded as effective ones. The study used non-probability sampling technique for the sample selection. All respondents are managers and engineers who have a thorough understanding of the enterprise and the industry. The manufacturers involved are mainly located in Hubei, Shanghai, Beijing, Chongqing, Guangzhou and Tianjin. Additionally, 90% of respondents have distributed innovation as a part of their enterprise strategy. In summary, samples are widely distributed and representative, thus meeting the requirements.

## 4. Results and discussion

### 4.1. Reliability analysis

In order to ensure the validity of this study, validity and reliability of samples shall be verified before data analysis. In this study, validity and reliability of samples were verified by factor analysis and internal consistency test, respectively. As shown in Table 1, Cronbach’s α coefficients of all measuring variables exceed 0.7, exhibiting good scale reliability. Data analysis by KMO and Bartlett’s test reveals that variables are suitable for factor analysis as the KMO value is 0.797 and the Chi-Square significance is 0.000. The accumulated factor contribution rates of measuring variables are no less than 0.652, indicating good validity of the scale. In summary, the proposed questionnaire scale shows high reliability and validity.

**Table 1:** Results of factors analysis and reliability and validity test.

Variable	Measurement item no.	Measurement item	Statistics		Factor load coefficient	Contribution rate of principal component variance	Cronbach’s α
			Mean	SD			
Distributed structure embedment	(1)	Project modularized allocation	7.372	1.084	0.699	0.652	0.782
	(2)	Geographical distribution of the 4S shops	7.266	1.249	0.712		
Distributed cognition embedment	(1)	Cognitive learning by employees	7.161	1.220	0.724	0.730	0.806
	(2)	Cultural compatibility mechanism	7.457	1.125	0.718		
	(3)	Trust between employees	7.571	1.461	0.735		
Knowledge collaborative embedment	(1)	Knowledge openness	7.285	1.196	0.782	0.807	0.797
	(2)	Information understanding, communication and cooperation	7.672	1.287	0.723		
	(3)	Knowledge management mechanism	7.699	1.232	0.737		
R&D capability	(1)	Ratio of investment in R&D	7.121	1.470	0.715	0.785	0.831
	(2)	Patent quantity	6.919	1.513	0.782		
	(3)	R&D employees	7.043	1.412	0.797		
Innovative performances of China’s automobile industry	(1)	Product update	7.326	1.270	0.762	0.823	0.799
	(2)	Success rate of product innovation	7.294	1.391	0.741		
	(3)	Technical contents of new products	7.075	1.437	0.794		
	(4)	Market shares of new products	7.516	1.321	0.812		

KMO value = 0.797; Chi-Square significance = 0.000.

### 4.2. Testing of hypothesis

**Figure 2:**
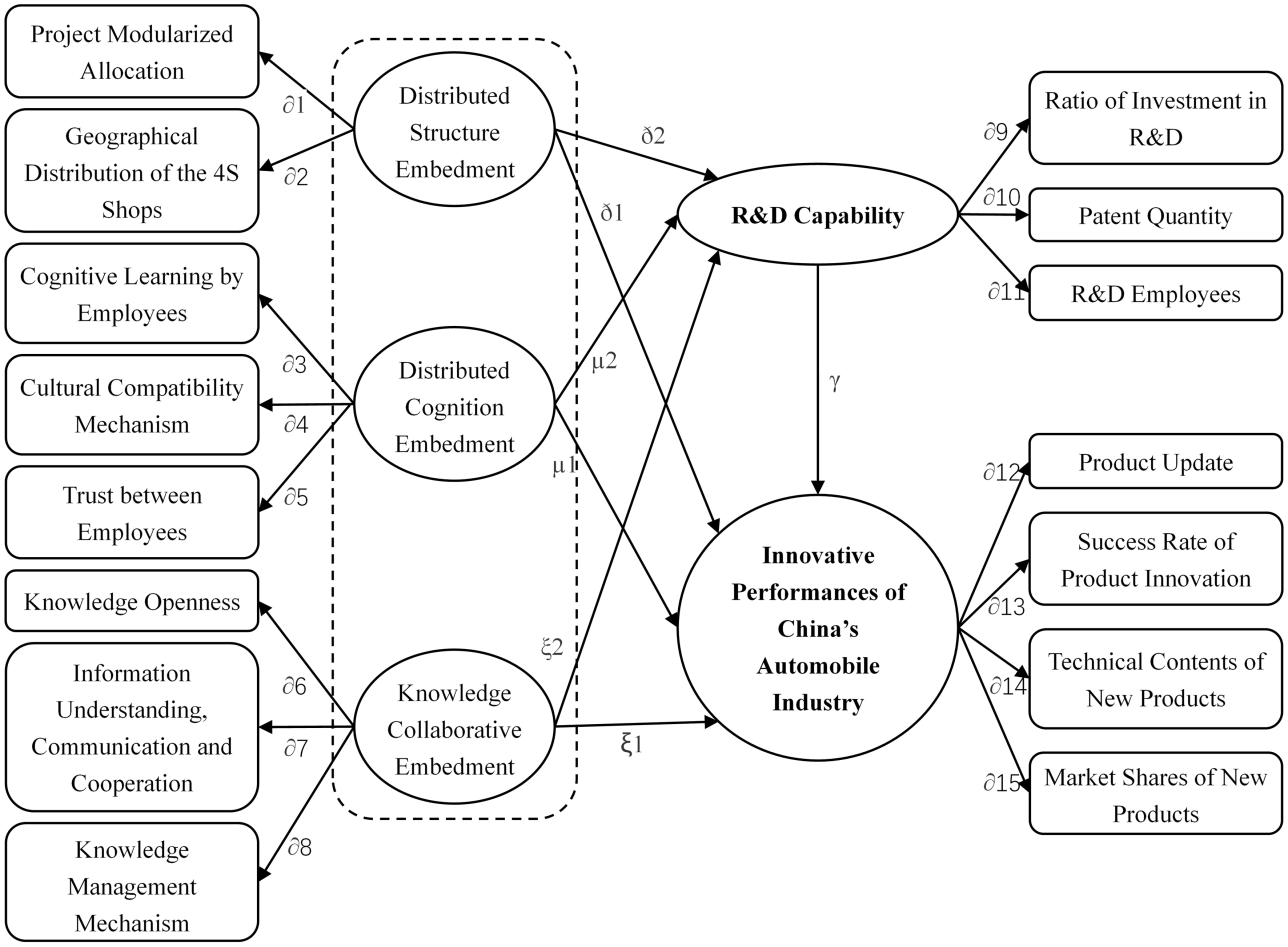
Estimated model.

Since reliability and validity of samples have been demonstrated to be reasonable, a theoretical model is proposed according to the hypotheses (see Figure 2) to investigate the correlations of variables. AMOS17.0 was used for verification as Coombs et al. (2003); Leonard-Barton (2003); Li and Jin (2010); Alexy and Leitner (2011); Liu et al. (2011); Peng (2012); Wencong and Guilong (2013); Osipitan (2014); Chen et al. (2017).

Model verifications of different variables demonstrate that fit indexes of the proposed theoretical model meet the requirements. Meanwhile, model verifications indicate that the goodness of fit index (GFI), the comparative fit index (CFI) and the root mean square error approximation (RMSEA) are 0.942, 0.973 and 0.035, all of which meet the requirements, demonstrating good fitting of the model and practical data. Hence, the results are statistically significant. Table 2 lists the results of hypothesis test. The path coefficients, corresponding hypotheses and *p* values in the theoretical model are summarized to obtain path coefficients and verification results.

**Table 2:** Results of hypothesis test.

Hypotheses	Path coefficients	Values	*p*-Value	Hypothesis label	Verification results
Distributed structure embedment → Innovative performances of China’s automobile industry	ð1	0.627	0.008	H1a	Valid
Distributed cognition embedment → Innovative performances of China’s automobile industry	μ1	0.538	0.013	H1b	Valid
Knowledge collaborative embedment → Innovative performances of China’s automobile industry	ξ1	0.653	***	H1c	Valid
Distributed structure embedment → China’s automobile industry R&D capability	ð2	0.613	0.004	H2a	Valid
Distributed cognition embedment → China’s automobile industry R&D capability	μ2	0.549	0.001	H2b	Valid
Knowledge collaborative embedment → China’s automobile industry R&D capability	ξ2	0.574	***	H2c	Valid
R&D capability → Innovative performances of China’s automobile industry	ɤ	0.691	***	H3	Valid

***denotes that *p* < 0.001.

As shown in Table 2, all path coefficients (ð, μ, ξ, ɤ) have statistical significances and Hypotheses H1a, H1b, H1c, H2a, H2b, H2c and H3 are valid. Hence, Hypotheses H1, H2 and H3 are valid, indicating that all hypotheses are verified. Additionally, hypothesis test demonstrates that distributed innovation embedment can influence innovative performances of China’s automobile industry directly and indirectly *via* R&D capability.

### 4.3. Discussion

Hypotheses H1a is valid. According to H1a, China’s automobile enterprises shall pay more attention to project modularized allocation and geographical distribution of 4S stores in order to increase their market shares.

Hypotheses H1b is valid. According to H1b, learning cognition and identification of corporate culture by employees have positive effects on innovative performances of China’s automobile industry.

Hypotheses H1c is valid. According to H1c, sharing, transfer and creation of key resources, namely knowledge, have significant effect on success rate and frequency of new product development and automobile industry shall pay more attention to knowledge management to develop intelligent enterprises and creative employees.

Hypotheses H2 is valid. According to H2, distributed innovation has positive effects on R&D capability of China’s automobile industry.

Hypotheses H3 is valid. According to H3, enhanced R&D capability has positive effects on technical content, success rate and frequency of new product development in China’s automobile industry.

In summary, China’s automobile enterprises shall enhance their innovative performances and competitiveness by participating in distributed innovation and improving R&D capability.

## 5. Conclusion

With the current situation of China’s automobile industry as the background, the correlation of distributed innovation embedment, R&D capability and innovative performance is investigated. Seven hypotheses are proposed and empirically verified based on practical data of China’s automobile industry. The results demonstrate that innovative performances of China’s automobile industry are highly dependent on distributed innovation embedment and R&D capability.

In terms of theory, the correlation of innovative performances in automobile industry, R&D capability and distributed innovation embedment is investigated from a microscopic perspective (i.e., distributed innovation embedment in China’s automobile industry) for the first time. Empirical and theoretical analyzes demonstrate positive correlations of these factors.

This study has multiple practical implication for the Chinese automobile industry and for the policy making authorities. First the study motivates the companies to engage their stakeholders in the decision-making process and motive the knowledge sharing from bottom to top. Secondly the management of the companies must manage the knowledge coming from different ends such as within the organization and outside the organization’s boundaries. The management of this information and manage the flow of the information is critical and challenging for the firms. The managers must stress on the need on the R& D especially in the new product development which resulted in the better financial performance of the companies. The management must promote the learning culture within organizations and promote the knowledge sharing among the employees. This study also facilitates the regulatory bodies by introducing and promoting the open innovation laws and support the firms practicing this approach. These practices will not only enhance the Chinese industry performance overall but also set the benchmark for the rest of the industries in China specifically and for the rest of growing economies in the neighbor generally. Additionally, this study provides references to improvements of innovative performances of China’s automobile industry. Automobile enterprises shall strengthen project modularization, trust between employees and knowledge collaboration. Meanwhile, automobile enterprises shall attach great importance to knowledge sharing and innovation management.

This study has several limitations. On the one hand, it is based on correlations of distributed innovation embedment, R&D capability and innovative performances of China’s automobile industry, while other factors affecting variable correlations have not been considered. On the other hand, the conclusions are only applicable to automobile industry in certain regions in China due to the insufficient sample size.

## Data availability statement

The raw data supporting the conclusions of this article will be made available by the authors, without undue reservation.

## Ethics statement

The studies involving human participants were reviewed and approved by the ethical committee of the Hubei University ref. 20/NSF/684201. The patients/participants provided their written informed consent to participate in this study.

## Author contributions

JL initiated the idea and conceptualize the idea, and wrote the initial draft. GL manage the data and collected the data, and validate the analysis. HC performed the data analysis. YC supervise the project and managed the resources and final document, and took supporting role in the data analysis. All authors contributed to the article and approved the submitted version.

## Funding

This project is funded by The Impact of Technology Lock-in on the Dynamic Upgrading of China’s Manufacturing Industry in Global Value Chain and Its Solution 20AGL006.

## Conflict of interest

The authors declare that the research was conducted in the absence of any commercial or financial relationships that could be construed as a potential conflict of interest.

## Publisher’s note

All claims expressed in this article are solely those of the authors and do not necessarily represent those of their affiliated organizations, or those of the publisher, the editors and the reviewers. Any product that may be evaluated in this article, or claim that may be made by its manufacturer, is not guaranteed or endorsed by the publisher.
